# Wildfire smoke and antimicrobial resistance: A hidden link demanding global attention

**DOI:** 10.1007/s13280-026-02382-2

**Published:** 2026-04-04

**Authors:** Ronan Adler Tavella, Fábio Parra Sellera, Daniela Debone, Simone Georges El Khouri Miraglia, Rodrigo Cayô, Nilton Lincopan, Ana Cristina Gales, Arnaldo Lopes Colombo, Sergio Schenkman, João Pedro Rueda Furlan

**Affiliations:** 1 Laboratory of Economics, Health and Environmental Pollution (LESPA), Institute of Environmental, Chemical and Pharmaceutical Sciences, Federal University of São Paulo, Rua São Nicolau, 210, Diadema, São Paulo 09913-030 Brazil; 2 Antimicrobial Resistance Institute of São Paulo (ARIES), São Paulo, Brazil; 3 School of Veterinary Medicine, Metropolitan University of Santos, Avenida Conselheiro Nébias – Boqueirão – Santos, São Paulo, 11045-002 Brazil; 4 Department of Internal Medicine, School of Veterinary Medicine and Animal Science, University of São Paulo, São Paulo, Brazil; 5 Laboratory of Environmental Antimicrobial Resistance (LEARN), Department of Biological Sciences (DCB), Institute of Environmental, Chemical and Pharmaceutical Sciences (ICAQF), Federal University of São Paulo (UNIFESP), Diadema, SP 09913-030 Brazil; 6 Department of Microbiology, Instituto de Ciências Biomédicas, University of São Paulo, Avenida Prof. Lineu Prestes, 1374 – Butantã, São Paulo, 05508-000 Brazil; 7 Department of Clinical Analysis, Faculty of Pharmacy, University of São Paulo, São Paulo, Brazil; 8 Department of Medicine, Division of Infectious Diseases, Federal University of São Paulo, Rua Botucatu, 740 – Vila Clementino, São Paulo, 04023-062 Brazil; 9 Escola Paulista de Medicina, Universidade Federal de São Paulo, Rua Botucatu, 740 – Vila Clementino, São Paulo, 04023-062 Brazil; 10 Department of Pharmaceutical Sciences, Health Sciences Center, Federal University of Paraíba, João Pessoa, Paraíba 58051-900 Brazil

**Keywords:** Air pollution, Fine particulate matter, Global Public Health, One Health, Superbugs

## Abstract

Wildfire smoke represents an underrecognized yet potentially important contributor to the dissemination of antimicrobial resistance (AMR). As wildfires increase in frequency and intensity due to climate change, they release vast amounts of PM_2.5_, particles with the potential to transport antimicrobial-resistant microorganisms and genetic elements across long distances. These severe air pollution events create multifactorial pressures on human and environmental health: increasing antimicrobial prescriptions through respiratory and systemic illnesses, overwhelming healthcare systems, and facilitating the environmental spread of multidrug-resistant strains through deposition in water, soil, and agri-food systems. This perspective highlights how wildfire-related PM_2.5_ may act as a direct vehicle and an indirect driver of AMR propagation across One Health domains. We emphasize the urgent need to integrate AMR surveillance into air quality monitoring, particularly in vulnerable regions. Understanding this possible relationship is essential for informing future research priorities, guiding public health strategies, and advancing interdisciplinary approaches to address these converging global health threats.

## Introduction

Air pollution and antimicrobial resistance (AMR) are significant global health concerns resulting in high morbidity and mortality rates worldwide. As a result, both crises add to the global disease burden, emphasizing their importance in shaping health outcomes (Ye et al. 2022; GBD 2021 Risk Factors Collaborators 2024; GBD 2021 Antimicrobial Resistance Collaborators 2024). Among air pollutants, particulate matter with an aerodynamic diameter of 2.5 µm or smaller (PM_2.5_) is the most detrimental to human health and represents the leading environmental risk factor for global mortality (GBD 2021 Risk Factors Collaborators 2024; Health Effects Institute 2025). Concurrently, wildfires have increased in frequency and intensity in multiple regions under a changing climate, becoming a major episodic source of PM_2.5_ and resulting in extreme air pollution events that extend far beyond their ignition sites (Kelley et al. 2025). Recently, the large-scale fires across Australia, Brazil, Canada, Chile, Portugal, and the USA have produced elevated PM_2.5_ concentrations, prolonged population exposures, and cross-border smoke transport, demonstrating the magnitude and global reach of wildfire-driven air pollution (Kelley et al. 2025; Kolden et al. 2025; Schollaert et al. 2025; Zhang et al. 2025a). Although there is evidence that increasing levels of air pollution are associated with an increased risk of AMR (Yan et al. 2022; Zhou et al. 2023, 2025), the role of wildfires remains a neglected aspect of planetary health.

## Wildfire smoke as a microbial and AMR carrier

Wildfire smoke is primarily composed of PM_2.5_, which accounts for about 90% of its total particle mass (United States Environmental Protection Agency 2025). The composition of this PM_2.5_ varies widely depending on combustion conditions and fuel type, but often includes a mixture of inorganic and organic compounds (Liu et al. 2019; Orr et al. 2025). Apart from their chemical constituents, wildland fires can aerosolize and emit microorganisms from burning biomass and underlying soils, entraining bacteria and fungi into the smoke plume, where they may be transported in association with fire-derived particulate matter (Kobziar and Thompson 2020; Kobziar et al. 2024; Bonfantine et al. 2024; Adhikari et al. 2026). Experimental and field evidence indicate that smoke-borne microbial assemblages contribute to the atmospheric aerobiome and can be traced back to terrestrial sources before being deposited into downwind ecosystems, reinforcing the role of wildfire smoke as a biological dispersal vector, a process increasingly examined within the emerging field of pyroaerobiology (Bonfantine et al. 2024; Kobziar et al. 2024; Ellington et al. 2024). In addition to direct emission from combusted materials, strong convective updrafts generated by large fires may draw microorganisms from surrounding soils and vegetation into the plume, further increasing its biological load (Kobziar and Thompson 2020). Notably, some microbial cells and fungal spores are within the aerodynamic size range of PM_2.5_, allowing them to exist as individual airborne particles or to associate with other particulate aggregates (Wei et al. 2019; Shammi et al. 2021; Gao et al. 2023; Ziaei et al. 2025).

Wildfire smoke may contain higher concentrations of microorganisms compared to background air, with field studies reporting microbial cell concentrations approximately fourfold to fivefold higher in smoke than in ambient air and an estimated 70–80% of cells remaining viable during emission and transport (Moore et al. 2021; Kobziar et al. 2022; Adhikari et al. 2026). Furthermore, PM_2.5_ has been identified as a carrier of antimicrobial-resistant bacteria (ARBs) and a facilitator of horizontal AMR gene transfer (Xie et al. 2019; Zhou et al. 2021; Kormos et al. 2022; Gao et al. 2023), raising urgent questions about its role in the dissemination, maintenance, and evolution of AMR. In this context, while there is currently no direct evidence linking PM_2.5_ to antifungal resistance, it is biologically plausible that wildfire smoke may harbor airborne fungi carrying resistance phenotypes.

Although many smoke-borne microorganisms are predominantly derived from natural biomass and soils, these reservoirs can still contain resistance determinants as part of the intrinsic environmental resistome (D’Costa et al. 2011), and smoke transport can redistribute these traits across ecosystems (Bonfantine et al. 2024; Adhikari et al. 2026). In addition, smoke plumes frequently interact with agricultural, peri-urban, and urban air masses (Singh et al. 2012; Jaffe et al. 2020), where PM_2.5_ is already known to harbor antimicrobial resistance genes (ARGs) and ARBs, providing a potential pathway for wildfire-derived particles to co-transport resistance signatures from both environmental and anthropogenic sources. Similarly, soil- and vegetation-associated fungal spores can be efficiently entrained and dispersed within fire plumes under intense convective dynamics, potentially enabling the broader atmospheric redistribution of environmentally antimicrobial-resistant strains. The atmospheric suspension of these biological particles can enhance their dispersal range and persistence in different niches (Després 2012; Xie et al. 2021).

In addition to its biological load, wildfire-derived PM_2.5_ also contains a complex mixture of chemical pollutants that can influence microbial survival and resistance dynamics. Airborne contaminants, such as heavy metals and polycyclic aromatic hydrocarbons, may exert selective pressure on microbial populations, co-selecting for AMR traits and favoring the persistence of antimicrobial-resistant microorganisms in atmospheric and deposited environments (Dickinson et al. 2019; Murray et al. 2024; Xiu and Hu 2025).

Recent large-scale analyses have demonstrated a robust association between PM_2.5_ concentrations and the prevalence of AMR at a global level. Zhou et al. (2023) analyzed data from over 100 countries and found that increases in PM_2.5_ were significantly correlated with rising clinical AMR rates across multiple bacterial pathogens, including *Klebsiella pneumoniae*. Notably, this relationship persisted even after accounting for confounding factors, including antimicrobial consumption and socioeconomic variables, suggesting that air pollution itself acts as an independent environmental driver of AMR. Moreover, this study estimated that a 10% reduction in PM_2.5_ could potentially decrease global AMR by approximately 17%, underscoring the profound health implications of airborne particles beyond traditional respiratory hazards. These findings reinforce the hypothesis that particulate matter, particularly PM_2.5_, may act as an environmental reservoir and transport vector for AMR determinants. However, it is important to recognize that most epidemiological evidence linking PM_2.5_ and AMR derives from chronically polluted urban environments, where particulate composition is largely shaped by traffic emissions, industrial activities, and other anthropogenic sources. Wildfire-derived PM_2.5_ differs substantially in its physicochemical profile, organic carbon content, combustion by-products, and associated biological material and microorganisms. As a result, the origin and ecological dynamics of ARGs and antimicrobial-resistant microorganisms may differ between urban pollution and wildfire smoke. Nevertheless, given the documented viability and long-range transport of smoke-associated microorganisms, together with evidence that a substantial fraction of these cells remains viable and is transported over long distances, wildfire-derived PM_2.5_ may exert similar or even amplified effects during acute pollution events, thereby representing a previously neglected contributor to AMR dissemination.

## Extreme air pollution events and One Health implications

Unlike regular urban air pollution, wildfire smoke releases massive amounts of PM_2.5_ in a short period, potentially exacerbating the airborne spread of AMR factors, including antimicrobial-resistant microorganisms, ARGs, and AMR-related mobile genetic elements (MGEs), within the One Health and Global Health perspectives. Due to their scale, wildfire smoke plumes are transported over vast distances by meteorological conditions, affecting air quality in urban, rural, and coastal areas hundreds or even thousands of kilometers away, exacerbating particulate matter levels, and triggering severe air pollution episodes (Hung et al. 2021; Kelley et al. 2025; Orr et al. 2025). These events often persist for days or weeks, leading to prolonged exposure and widespread public health impacts (Reid et al. 2016; Aguilera et al 2021; Zhang et al. 2025a). Historical instances, including the 2019–2020 Australian bushfires (Graham et al. 2021), the 2020 extreme wildfire season in the USA (Higuera and Abatzoglou 2020), followed by major outbreaks such as those in Maui (Hawaii) in 2023 (Mass and Ovens 2024) and California in 2025 (Schollaert et al. 2025), the 2023 Canadian wildfire season (Zhang et al. 2025a), the 2024 crises in Brazil and Chile (Miraglia et al. 2025a; Kolden et al. 2025), and the 2025 episodes in Portugal (Miraglia et al. 2025b), illustrate the scale of these episodes, during which PM_2.5_ concentrations soared to multiples of air quality thresholds and population exposures were sustained across multi-day periods.

## Pathways from air pollution to AMR in humans

Episodes of extreme air pollution, such as those triggered by wildfires, may increase AMR through multiple pathways. One of the main concerns is their impact on human health and antimicrobial consumption. Exposure to high levels of PM_2.5_ is well documented to cause respiratory and cardiovascular distress (World Health Organization 2021; Health Effects Institute 2025) and has been associated with increased risk of bacterial pneumonia and coccidioidomycosis (Reid et al. 2019; Mulliken et al. 2023). Beyond these immediate health outcomes, large wildfire events have been shown to substantially increase hospital admissions and mortality rates. For instance, Zhang et al. (2025a) demonstrated that the 2023 Canadian wildfires contributed to thousands of acute and chronic deaths across North America and Europe, while Requia et al. (2021) found a ~ 23% rise in respiratory hospitalizations in Brazil during wildfire-related air pollution exposure. In addition, long-term exposure to PM_2.5_ was found to be strongly associated with cancers (Sundas et al. 2024). Accordingly, these conditions lead to increased hospital admissions and the risk of AMR-related infections (Mahendran et al. 2025).

Consequently, these acute health burdens related to wildfire smoke exposure and air pollution often drive increased healthcare seeking and antimicrobial consumption/perceptions (Abelenda-Alonso et al. 2024), especially when pollution-induced symptoms mimic or trigger infections, such as pneumonia, acute bronchitis, coccidioidomycosis, and other acute upper and lower respiratory tract infections (Reid et al. 2016; Mulliken et al. 2023; Zhang et al. 2025b). In addition to the individual antimicrobial consumption, the burden on healthcare systems creates conditions conducive to the transmission of AMR. Indeed, healthcare settings have been hotspots for amplification of AMR, and during severe air pollution episodes, the combination of weakened and overloaded systems, increased antimicrobial prescriptions, and higher patient turnover may intensify the spread of AMR. These issues may increase the antimicrobial selection pressure on microbial populations and accelerate the occurrence of multidrug-resistant microorganisms, also known as superbugs.

## Environmental dissemination of AMR via wildfire smoke

From an environmental perspective, wildfire smoke may act as a vehicle that introduces superbugs and MGEs related to AMR into different ecosystems, impacting not only humans but also livestock and wildlife populations. Runoff from fire-impacted areas may further accelerate the dissemination of superbugs into irrigation systems, livestock water supplies, and food production chains, amplifying the emergence of AMR. Once deposited in rural areas, wildfire-derived particles may also spread AMR factors into water bodies and crops, influencing microbial communities in agri-food systems. However, additional studies are required to determine whether and to what extent this pathway contributes to AMR dissemination.

Emerging evidence has revealed that air pollutants do not act solely as passive carriers but actively shape the atmospheric resistome. Gao et al. (2023) demonstrated that PM_2.5_ and associated chemical pollutants strongly influence the abundance, diversity, and mobility of ARGs in the atmosphere. Their analysis showed that samples with higher particulate contamination exhibited increased ARG loads, and that certain atmospheric pollutants were significantly correlated with enriched resistome profiles, suggesting mechanisms of co-selection and stabilization of ARGs on MGEs. Although these findings were derived primarily from non-wildfire particulate pollution, where sources and composition differ from wildfire smoke, the underlying mechanisms, such as pollutant-mediated co-selection and particle-associated gene stabilization, may also operate during intense wildfire events. In this context, wildfire smoke could generate episodic pulses of atmospheric resistome enrichment, potentially intensifying the dissemination of ARGs across interconnected environmental compartments.

## Expansion into wildlife and aquatic systems

**Fig. 1:**
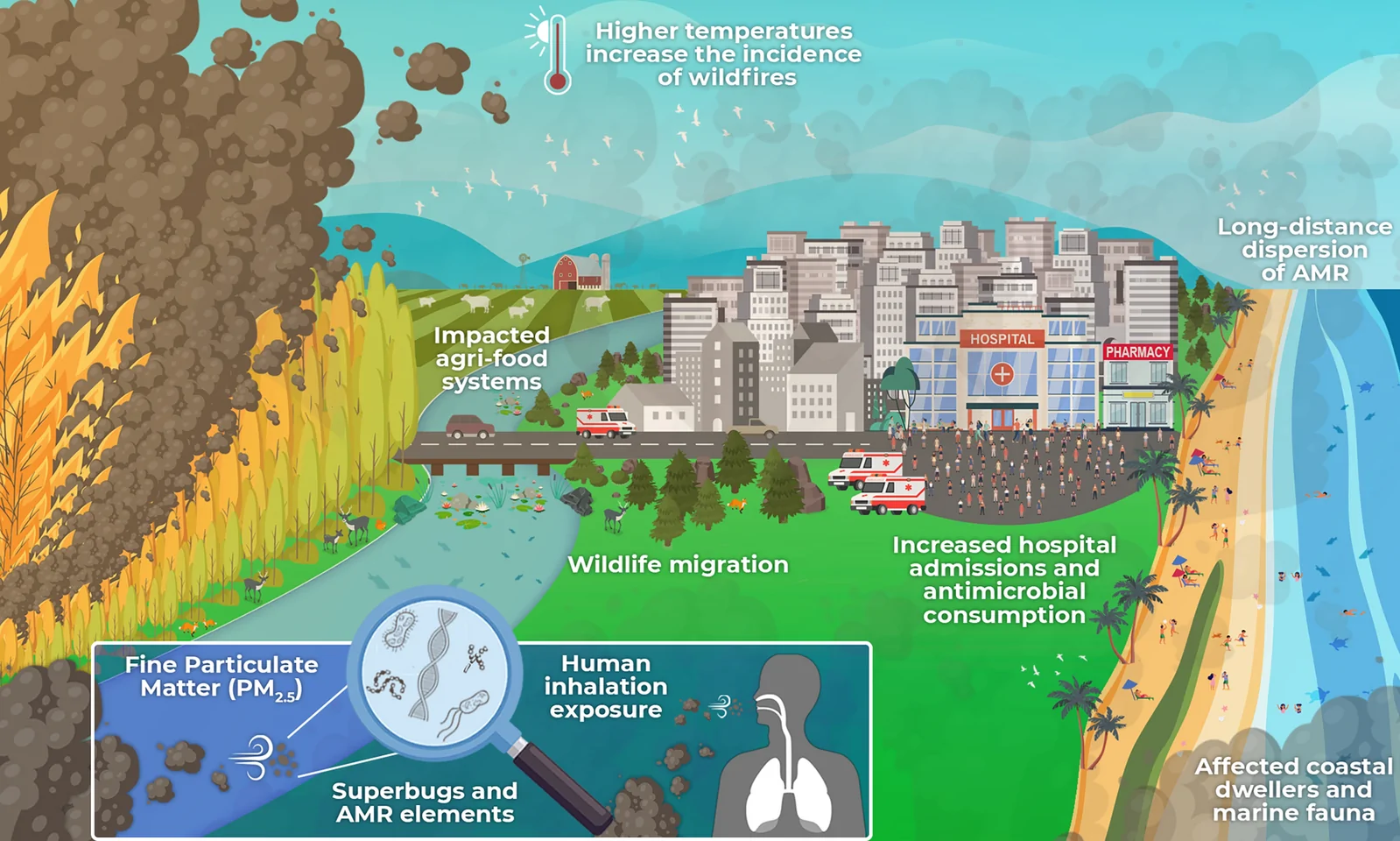
The potential role of wildfire smoke in the emergence of AMR. In addition to overwhelming health care facilities, the dispersion and deposition of PM_2.5_ related to wildfire smoke may favor the spread of AMR and selects of superbugs in urban, rural, terrestrial, and coastal environments. Figure created with vectors from Freepik.com

The exposure of natural environments to wildfire smoke containing antimicrobial-resistant microorganisms and/or AMR factors may introduce these elements into terrestrial and aquatic ecosystems beyond the immediate burn area. Following plume dispersion, particle settling and wet deposition may incorporate these microbial constituents into soils, vegetation, freshwater systems, and nearshore environments. In fire-affected watersheds, post-fire runoff and erosion may further mobilize deposited particles into rivers, estuaries, and coastal zones, creating additional pathways for environmental dissemination. Coastal cities exposed to these pollutants may therefore experience secondary contamination of marine environments, potentially affecting coastal dwellers and marine animals (Zhu et al. 2017; Serrana et al. 2025). The situation in nearshore regions could be aggravated by the exposure of migratory wild animals and natural events, such as ocean winds and currents, which may contribute to the long-distance dispersion of AMR and further spillover into preserved natural areas and inhospitable regions (Martins et al. 2018). This process illustrates how wildfire-driven air pollution extends the reach of AMR beyond immediate impacts, transcending geographical and ecological boundaries (Fig. 1).

## Conclusion

Despite the growing global burden of AMR and the increasing frequency and intensity of wildfires driven by climate change, wildfire-related severe air pollution events remain an overlooked dimension in understanding the environmental pathways of AMR dissemination, especially in low- and middle-income countries. These extreme events require integration of air quality monitoring with phenotypic, molecular, and genomic surveillance of AMR to determine whether wildfire smoke amplifies this silent crisis. In low- and middle-income settings, this could be supported through the combination of satellite-based smoke tracking, expansion of ground-level air quality monitoring (including low-cost sensors), and strengthened laboratory surveillance networks capable of detecting resistance determinants in both clinical and environmental samples. Hence, addressing this issue calls for interdisciplinary research along with urgent and integrated strategies that reduce emissions, improve antimicrobial stewardship, and strengthen environmental and health policies. Bridging the air pollution and AMR agendas will be essential to mitigate the escalating threat of AMR propagation in an era of intensifying climate-driven disasters.

## Data Availability

This manuscript uses only publicly available data that has been appropriately cited.
